# DNA Sensor IFI204 Contributes to Host Defense Against *Staphylococcus aureus* Infection in Mice

**DOI:** 10.3389/fimmu.2019.00474

**Published:** 2019-03-18

**Authors:** Wei Chen, Shui-Xing Yu, Feng-Hua Zhou, Xiao-Jing Zhang, Wen-Ying Gao, Kun-Yu Li, Zhen-Zhen Liu, Wen-Yu Han, Yong-Jun Yang

**Affiliations:** ^1^Key Laboratory of Zoonosis Research, Ministry of Education, College of Veterinary Medicine, Jilin University, Changchun, China; ^2^State Key Laboratory of Reproductive Regulation and Breeding of Grassland Livestock, School of Life Sciences, Inner Mongolia University, Hohhot, China; ^3^Institute of Translational Medicine, The First Hospital, Jilin University, Changchun, China

**Keywords:** *Staphylococcus aureus* (MRSA), IFI204, DNA sensor, STING, IFI16, innate immune, phagocytosis, extracellular trap

## Abstract

Interferon-inducible protein (IFI204) (p204, the murine homolog of human IFI16) is known as a cytosolic DNA sensor to recognize DNA viruses and intracellular bacteria. However, little is known about its role during extracellular bacterial infection. Here we show that IFI204 is required for host defense against the infection of *Staphylococcus aureus*, an extracellular bacterial pathogen. IFI204 deficiency results in decreased survival, increased bacterial loads, severe organs damage, and decreased recruitment of neutrophils and macrophages. Production of several inflammatory cytokines/chemokines including IFN-β and KC is markedly decreased, as well as the related STING-IRF3 and NF-κB pathways are impaired. However, exogenous administration of recombinant KC or IFN-β is unable to rescue the susceptibility of IFI204-deficient mice, suggesting that other mechanisms rather than KC and IFN-β account for IFI204-mediated host defense. IFI204 deficiency leads to a defect in extracellular bacterial killing in macrophages and neutrophils, although bacterial engulf, and intracellular killing activity are normal. Moreover, the defect of bactericidal activity is mediated by decreased extracellular trap formation in the absence of IFI204. Adoptively transferred WT bone marrow cells significantly protect WT and IFI204-deficient recipients against *Staphylococcus* infection compared with transferred IFI204-deficient bone marrow cells. Hence, this study suggests that IFI204 is essential for the host defense against *Staphylococcus* infection.

## Introduction

The pathogen *Staphylococcus aureus* persistently colonizes a large proportion of the human population and is a frequent cause of skin and soft tissue infections, pneumonia, and sepsis. Despite intense research in understanding the pathogenesis and host-pathogen interaction, the mechanisms by which *Staphylococcus* is cleared from the host are largely unclarified, thereby impeding the development of novel strategies for control of this infection.

The innate immune system plays a key role in the early recognition and elimination of invading pathogens. Sensing bacteria through pattern recognition receptors (PRRs) enables innate immune cells to categorize microbial invaders and to initiate appropriate signaling cascades that mobilize defense mechanisms (1). Characterized as a DNA sensor, interferon-inducible protein 204 (IFI204) (its human ortholog IFI16) is one member of PRRs that detects cytosolic DNA for the type I IFN response (2). In response to cytosolic DNA stimulation or virus infection, IFI204/IFI16 interacts with STING to induce TBK1-dependent IFN-β production. Several studies also reported that IFI204/IFI16 recognizes DNA viral genomes in the nucleus and activates the inflammasome pathway through ASC and caspase-1, leading to IL-1β, and IL-18 production (3, 4).

IFI204/IFI16 is also implicated in sensing intracellular bacterial infection. Knockdown of IFI204/IFI16 by small interfering RNA significantly inhibited IFN-β release in response to intracellular bacterial infections such as *Francisella novicida* (5), *Listeria monocytogenes* (6), *Mycobacterium bovis* (7). Previously, we also showed that *Listeria monocytogenes*-derived genomic DNA triggered programmed cell death in human trophoblasts via IFI16 (8). Obviously, cytosolic bacteria-derived dsDNA is the stimulating ligand for IFI204/IFI16-mediated immune responses.

In contrast to the extensive studies of IFI204/IFI16-mediated response to viral and intracellular bacterial infections through gene knockdown *in vitro*, there is little knowledge regarding the role of IFI204/IFI16 in response to extracellular bacterial infection. Here, using IFI204-deficient mice we explored the action of IFI204 in host defense against *Staphylococcus*, which is generally spoken as an extracellular bacteria.

We find that IFI204 protects the host against *Staphylococcus* infection. IFI204-deficient mice exhibit higher mortality rates, more bacterial loads, and severer organs damage compared with control mice. Although IFI204 deficiency results in a defect of IFN-β and KC production through impairing STING-IRF3 and NF-κB signaling, neither IFN-β nor KC accounts for IFI204-mediated host defense. IFI204 deficiency inhibits extracellular bacterial killing rather than engulf and intracellular killing activities. Interestingly, we find that the defect of bactericidal activity in the absence of IFI204 is mediated by decreased extracellular trap formation. Collectively, our results suggest that IFI204 is essential for the host defense against extracellular bacterial infection through enhancing bactericidal activity.

## Materials and Methods

### Mice and Cells

IFI204-deficient mice were purchased from Nanjing Biomedical Research Institute of Nanjing University (Nanjing, China) and were subsequently backcrossed onto the C57BL/6J background for another eight generations. Heterozygous breeding pairs were used to generate wild-type (WT) mice. Bone marrow-derived macrophages (BMDMs) were isolated from mouse femurs of 8–10 week old mice and cultured in RPMI1640 medium containing 10% heat-inactivated FBS, 25% L929 cell–conditioned medium, 100 U/mL penicillin, and 100 U/mL streptomycin at 37°C in a humidified atmosphere containing 5% CO_2_. Cells were harvested for assays at day 7 of differentiation. For isolation of elicited peritoneal macrophages (PMs), age- and sex-matched WT and IFI204-deficient mice were intraperitoneally (i.p.) injected with 1.0 mL of 3% sterile thioglycollate broth (Sigma-Aldrich). Four days after the injection, cells were harvested by i.p. lavage with ice-cold PBS and cultured in DMEM medium containing 10% heat-inactivated FBS.

### Pulmonary and Systemic Infection

*Staphylococcus* USA300 strain was grown to exponential phase in Tryptic Soy Broth (TSB) at 37°C. Six to eight weeks old sex-matched mice were intranasally or intravenously infected with 1 × 10^8^ or 2 × 10^8^ colony-forming unit (CFU) *Staphylococcus* USA300 diluted in PBS in a total volume of 20 or 200 μL. Bronchoalveolar lavage fluid (BALF) was obtained by lavaging the lung with 1 mL PBS containing 100 μg/mL soybean trypsin inhibitor.

### Bacterial Burden and Cytokine Measurements

Aseptically excised tissues were homogenized. Serial dilutions of tissue homogenates were plated on agar plates and bacterial loads (CFU/g) were determined by colony counting after overnight incubation. The tissues were homogenized mechanically in cold PBS (at a ratio of 6 mL per gram tissue) containing complete protease inhibitor cocktail and 1% Triton X-100. Tissue homogenates were then centrifuged at 12,000 rpm for 20 min. The supernatants were collected. Concentrations of various cytokines/chemokines in BALF, tissue homogenates or cell culture supernatants were determined by ELISA using antibody pairs from R&D according to manufacturer's manual.

### Tissue Histology and Immunostaining

Tissue samples of lung and kidney were fixed in buffered formalin solution (4%) and embedded in paraffin. Tissue sections (5 μm) were deparaffinized, rehydrated, and stained with hematoxylin-eosin. For immunohistochemistry, sections were subjected to an antigen retrieval step, followed by blocking for 1 h at room temperature, then stained with IFI204 (Lifespan), Ly-6G/Ly-6c (BioLegend), and F4/80 (BioLegend) antibodies. Subsequently, specific staining was detected using the UltraSensitive S-P Kit and DAB Detection Kit (Maixin-Bio) according to the manufacturer's directions. For immunofluorescence, cells were stained with phospho-IRF3 (Santa Cruz), IFI204 (Lifespan), STING (Proteintech) primary antibodies, and Alexa Fluor® 488-conjugated secondary antibodies (Invitrogen). Kidney cell apoptosis was analyzed by TUNEL staining using a commercial kit (KeyGEN Biotech). DAPI (1 μg/mL) was used to stain nuclei.

### Quantitative PCR

RNA was isolated using TRI reagent (Sigma-Aldrich) and converted into cDNA. Subsequently, Real-Time PCR assays were performed using SYBR Green (Roche) on ABI Prism 7500 sequence detection system (Applied Biosystems). Gene expression levels were calculated using the 2^−Δ*Ct*^ method. The following primers were used: IFN-β sense 5′-ACT GCC TTT GCC ATC CAA GA-3′, antisense 5′-CAC TGT CTG CTG GTG GAG TT-3′. KC sense 5′-ACC CTG AAG CTC CCT TGG TT-3′, KC antisense 5′-AGA AGC CAG CGT TCA CCA GA-3′. IFI204 sense 5′-CAG GGA AAA TGG AAG TGG TG-3′, IFI204 antisense 5′-CAG AGA GGT TCT CCC GAC TG-3′. GAPDH sense 5′-CAC CCC AGC AAG GAC ACT GAG CAA G-3′, antisense 5′-GGG GGT CTG GGA TGG AAA TTG TGA G-3′.

### Western Blotting

The cells or tissues were homogenized in lysis buffer solution (1% Triton X-100, 50 mM Tris-HCl, 150 mM NaCl, 0.1 mM Na_3_VO4) supplemented with complete protease inhibitor cocktail (Sigma-Aldrich). The lysates were separated by SDS-PAGE, and transferred onto PVDF membrane. The membranes were blotted with antibodies against IFI204 (Lifespan), phospho-IRF3 (Santa Cruz), phospho-IκBα (Cell Signaling Technology), IκBα (Cell Signaling), phospho-NF-κB P65 (Cell Signaling), IRF3 (Abcam), IFI204 (Lifespan), STING (Proteintech), GAPDH (Proteintech), or β-Tubulin (Sungene Biotech).

### Administration of Recombinant KC and IFN-β

IFI204^−/−^ mice were i.p. injected recombinant KC or IFN-β (MBL International) at a dose of 1.0 μg per mouse in 100 μL PBS on Day −1 and Day 0. The mice were infected intranasally with 1 × 10^8^ CFU of *Staphylococcus* on Day 0. Aseptically excised tissues were homogenized at 24 hpi. Serial dilutions of tissue homogenates were plated on agar plates and bacterial loads (CFU/g) were determined by colony counting after overnight incubation.

### *In vivo* Neutralization of IFNAR1

Mice were i.p. inoculated with 2.5 mg anti-mouse IFNAR1 neutralizing mAb (clone MAR1-5A3, BioXcell) or 2.5 mg IgG isotype control (Clone MOPC-21, BioXcell). Twenty-four hours later, the mice were anesthetized with pentobarbital sodium and i.v. challenged with 2 × 10^8^ CFU of *Staphylococcus* suspended in 200 μL PBS. Mortality was monitored.

### Internalization Assay

To determine whether IFI204 impact the bacterial internalization of *Staphylococcus*, 2.5 × 10^9^ CFU/mL live or heat-killed bacteria were incubated with 0.15 mg/mL fluorescein isothiocyanate (FITC) in the dark for 30 min at RT. The bacteria were washed 3 times with PBS to remove unbound FITC. WT or IFI204^−/−^ BMDM were treated with FITC-labeled live or killed bacteria (MOI = 5) for indicating times. The extracellular fluorescence was quenched using 0.2% trypan blue. The mean fluorescence intensity (MFI) of the FITC-positive cells were determined by flow cytometric analyses.

### Intracellular and Extracellular Killing Assays

To determine whether IFI204 impact the intracellular bacterial killing capacity of macrophages, WT or IFI204^−/−^ BMDM were incubated with *Staphylococcus* (MOI = 5) for 1 h, and then non-engulfed bacteria were killed with 100 μg/mL gentamicin for 1 h. The cells were lysed with 0.1% Triton X-100 and intracellular bacterial were enumerated by serial dilution and plating on TSB agar plates. To determine whether IFI204 impact the extracellular bacterial killing capacity of macrophages or neutrophils, WT and IFI204^−/−^ BMDM or neutrophil were incubated with *Staphylococcus* (MOI = 5) for 6 h, the supernatant was collected and centrifuged at 600 × g for 5 min. The pelleted bacteria were resuspended in PBS and plated on TSB agar plates to enumerate the extracellular bacteria.

### ETs Formation Assays

Bone marrow macrophages or neutrophils were isolated from WT or IFI204^−/−^ mice. The cells were seeded on 12-mm 0.01% poly-l-lysine–coated coverslips in 24 well-plates and were challenged with bacteria (MOI = 50). Cells were fixed with 4% paraformaldehyde and then stained with SYTOX Orange (5 μM) and Hochest 33342 (2 μM). ETs were visualized on a fluorescence microscope and images were taken. Macrophages were stimulated with bacteria (MOI = 50) and PMA (100 nM). SYTOX Orange was added after 6 h and fluorescence was measured by spectrofluorometry.

### Adoptive Transfer of Bone Marrow Cells

Six-eight week old mice were lethally irradiated with 10 Gy of γ radiation at a rate of 1.5 Gy/min in a ^137^Cs irradiator. Within 24 h of irradiation, mice received an intravenous injection of 8 × 10^6^ bone marrow cells harvested from the femurs and tibias of WT or IFI204^−/−^ mice. Mice were allowed to recover at least 7 weeks before being used for experiments. Efficient reconstitution by donor bone marrow cells was confirmed by PCR for the IFI204 gene in splenocytes (**Figure 8G**).

### MPO Assay

Lung tissues were homogenized in 0.5% cetyltrimethylammonium chloride (4 μL/mg lung). The cleared supernatant was used for MPO assay to determine the infiltration of neutrophils. Briefly, samples in duplicate (75 μL) were mixed with equal volumes of the substrate (3,3′, 5,5′-tetramethyl-benzidine dihydrochloride, 3 mmol/L; resorcinol, 120 μmol/L; and H_2_O_2_, 2.2 mmol/L) for 2 min. The reaction was stopped by adding 150 μL of 2 mol/L H_2_SO_4_. The OD was measured at 450 nm.

### Statistical Analysis

Date are represented as mean ± SEM. Differences between mean values of normally distributed data were assessed with one-way ANOVA (Dunnett's *t*-test) and two-tailed Student's *t*-test. Log-rank test was used for statistical analysis of animal mortality. ^*^p < 0.05; ^**^p < 0.01 compared with control group. Statistical analysis was performed using Prism (GraphPad Software, La Jolla, CA).

## Results

### IFI204 Deficiency Attenuates Bacterial Clearance Following *Staphylococcus* Pulmonary Infection

**Figure 1:**
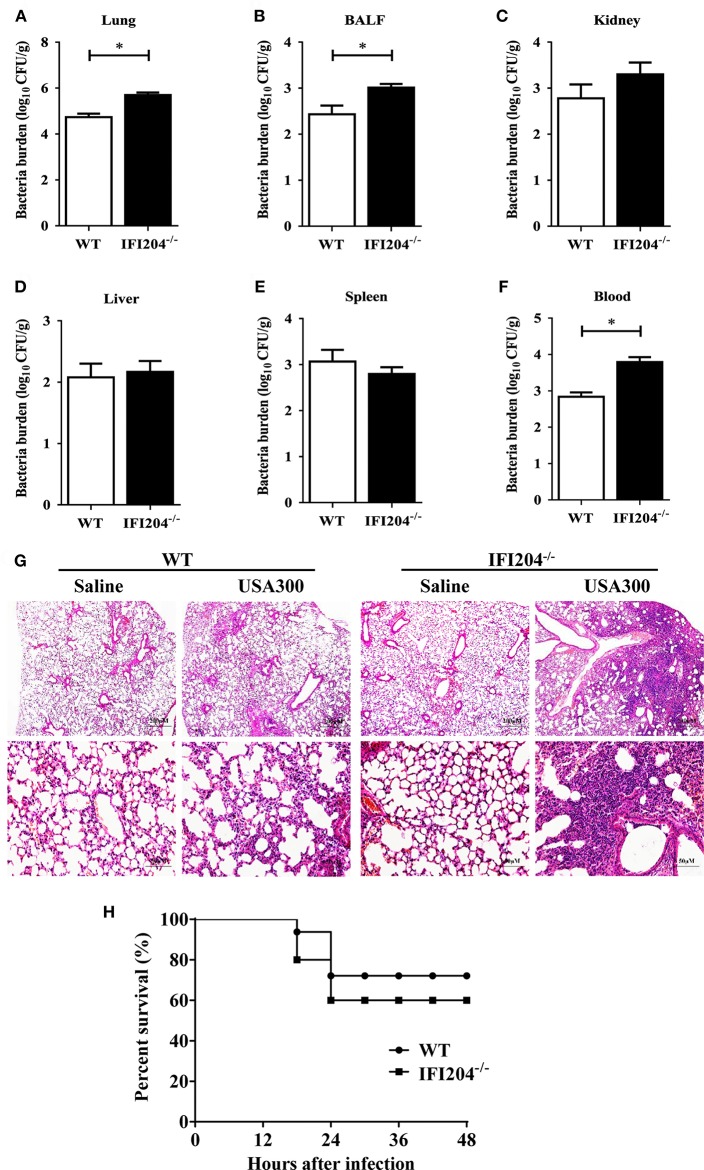
Interferon-inducible protein 204 (IFI204)-deficient mice display increased susceptibility to *Staphylococcus* pulmonary infection. **(A–F)** Age- and sex-matched WT (*n* = 8) and IFI204^−/−^ (*n* = 10) mice were infected intranasally with 1 × 10^8^ CFU of *Staphylococcus*. Homogenized tissues, BALF and blood were subjected to plating serial dilution for bacterial loads at 16 hpi. All data are shown as mean ± SEM. Student's *t*-test was performed. ^*^p < 0.05. **(G)** Hematoxylin-eosin staining of lung tissues collected from WT and IFI204^−/−^ (*n* = 5 each group) mice at 48 hpi. Magnification 40 × or 200 ×. **(H)** WT and IFI204^−/−^ mice were inoculated intranasally with 2 × 10^8^ CFU of *Staphylococcus* (*n* = 15) or PBS (*n* = 13). The animals were monitored every 6 h up to 48 h for survival.

Using *Staphylococcus* lung infection model, we initially explored the possible involvement of IFI204 in the host response to *Staphylococcus* infection. WT (wild-type) and IFI204^−/−^ (IFI204-deficient) mice were intranasally infected with 1 × 10^8^ CFU (colony-forming units) of *Staphylococcus*. Bacterial burdens were assessed in BALF (bronchoalveolar lavage fluid), blood and various tissues at 16 hpi by CFU counting. Significantly more bacteria were detected in the lungs, BALF and blood of IFI204^−/−^ mice compared with WT mice (Figures 1A–F). In line with this, histopathological examination of lung tissues 48 h after *Staphylococcus* challenge showed that there were more severe injury in IFI204^−/−^ mice (Figure 1G). To further characterize the role of IFI204 in host defense against *Staphylococcus*, WT and IFI204^−/−^ mice were intranasally infected with 2 × 10^8^ CFU of *Staphylococcus* and animal mortality was then monitored. IFI204^−/−^ mice demonstrated a slight lower survival rate than their WT counterparts within 48 hpi, but the difference was not significant (Figure 1H). Thus, these data suggest that IFI204 is involved in host defense against *Staphylococcus* pulmonary infection.

### IFI204 Deficiency Decreased Inflammatory Cells Recruitment and Cytokines/Chemokines Production

**Figure 2:**
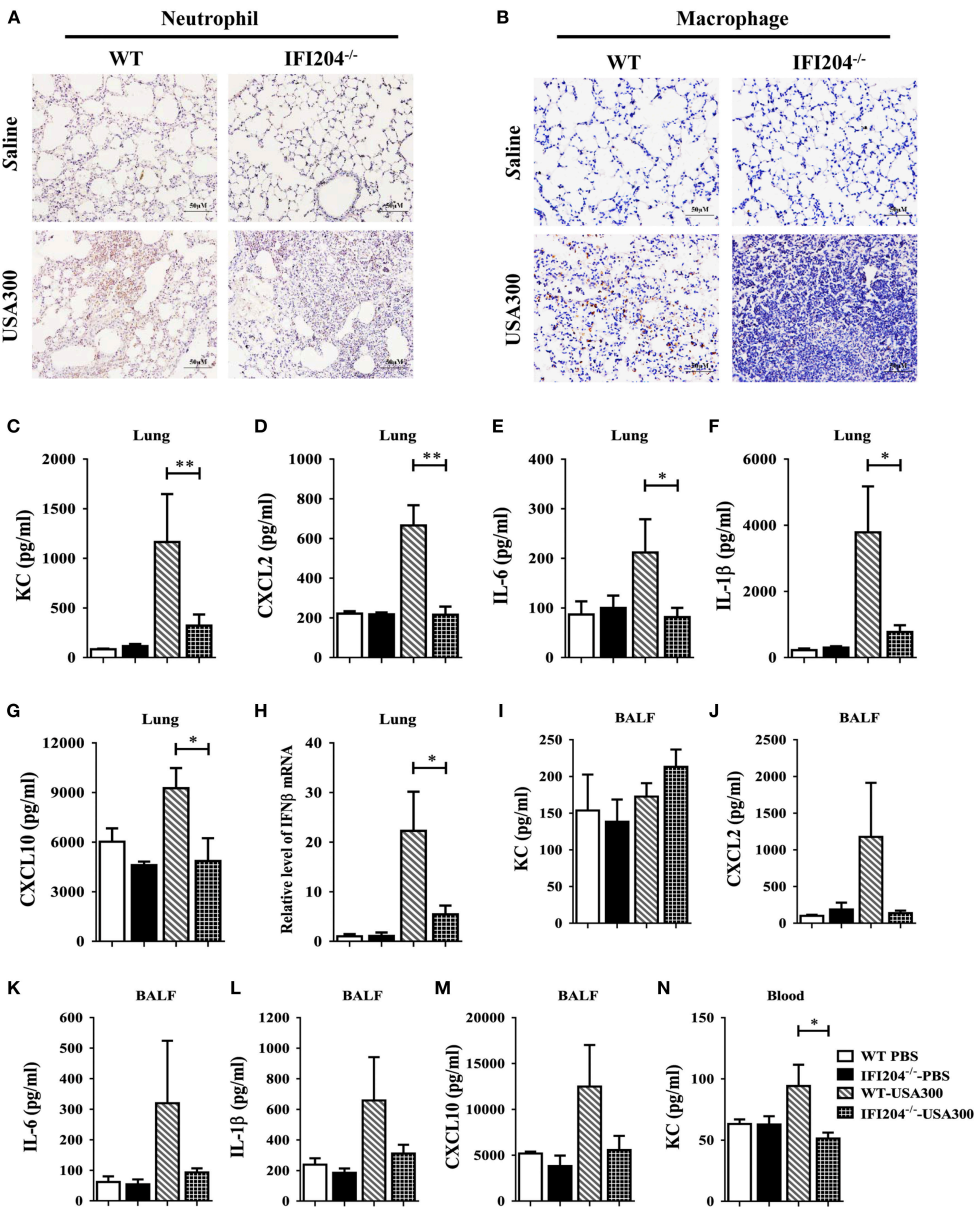
IFI204 is required for elevated inflammatory cells recruitment and cytokines/chemokines production after *Staphylococcus* pulmonary infection. WT and IFI204^−/−^ mice (*n* = 10 each group) were infected intranasally with 1 × 10^8^ CFU of *Staphylococcus* for 24 h. Representative immunohistochemical staining of **(A)** Gr-1 (a neutrophil marker) and **(B)** F4/80 (a macrophagocyte marker) were performed in the lung sections. **(C–N)** Levels of KC, CXCL2, IL-6, IL-1β, CXCL10, and IFN-β in lung, BALF or blood were determined. All data are shown as mean ± SEM. Student's *t*-test was performed. ^*^p < 0.05; ^**^p < 0.01.

To identify the potential mechanisms that contribute to higher bacterial loads in IFI204^−/−^ mice, we examined lung recruitment of neutrophil and macrophage because these cells are critical for the clearance of bacteria. Histological study showed less neutrophils and macrophages accumulation in the airways of IFI204^−/−^ mice at 24 hpi compared with WT mice (Figures 2A,B). In mice receiving the sham-operation, no significant cellular influx was observed in the lung of both genotype mice. To determine if the decreased inflammatory cells influx is dependent on inferior production of cytokines/chemokines following *Staphylococcus* infection, we measured the expression of cytokines (IL-6, IL-1β, and IFN-β) and chemokines (KC/CXCL1, CXCL2, and CXCL10) in lung homogenates, BALF or blood at 24 h after *Staphylococcus* challenge (Figures 2C–N). Bacterial challenge dramatically induced the release of these cytokines/chemokines from WT mice. However, the production of these cytokines/chemokines in IFI204^−/−^ mice were attenuated compared with WT mice. Thus, IFI204 deficiency results in impaired immune responses to *Staphylococcus* pulmonary infection.

### IFI204 Deficiency Attenuates Bacterial Clearance Following *Staphylococcus* Systemic Infection

**Figure 3:**
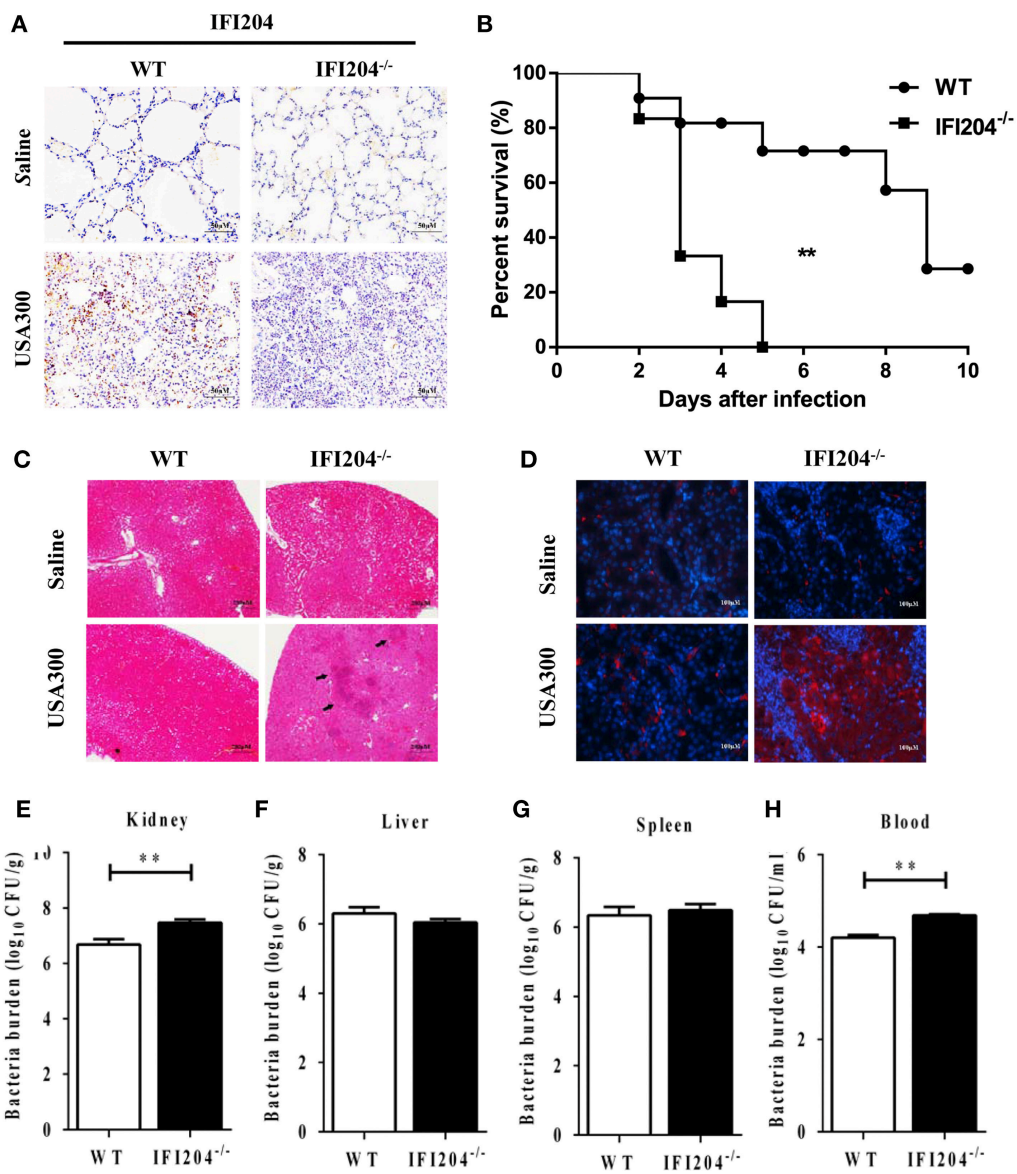
IFI204-deficient mice displayed increased susceptibility to *Staphylococcus* systemic infection. **(A)** Lung sections were stained with anti-IFI204. **(B)** WT (*n* = 15) and IFI204^−/−^ (*n* = 13) mice were infected *i.v* with 1 × 10^8^ CFU of *Staphylococcus*. The animals were monitored daily up to 10 days for survival. The Kaplan-Meier and log-rank methods were used to analyze survival rates. **(C,D)** Kidney tissues were collected from WT and IFI204^−/−^ mice at 48 hpi (*n* = 5 each group). Kidney injury was assessed by hematoxylin-eosin staining **(C)** and terminal deoxynucleotidyl transferase dUTP nick end labeling (TUNEL) histology **(D)**. Representative results are depicted. **(E–H)** Bacterial loads in kidney, liver, spleen, and blood of WT (*n* = 8) and IFI204^−/−^ (*n* = 10) at 20 hpi were determined. All data are shown as mean ± SEM. Student's *t*-test was performed. ^**^p < 0.01.

To further characterize the role of IFI204 in pulmonary defense against *Staphylococcus*, the expression of IFI204 in the lung sections was investigated by immunohistochemical staining. Our results showed that IFI204 staining was mainly detected in the recruited inflammatory cells of the infected lungs (Figure 3A). Next, we asked if IFI204 regulated host defense against *Staphylococcus* systemic infection. WT and IFI204^−/−^ mice were i.v. infected with 1 × 10^8^ CFU of *Staphylococcus*. Animal mortality was then monitored for 10 d. IFI204^−/−^ mice demonstrated a significantly lower survival rate than their WT counterparts (Figure 3B). Kidney injury was more severe in IFI204^−/−^ mice compared with WT mice as determined by H&E and TUNEL histology (Figures 3C,D). To determine whether a defect in bacterial clearance contribute to the death of IFI204-deficient mice, bacterial numbers in blood, spleen, kidney, and liver were enumerated at 24 hpi. As compared with WT mice, IFI204-deficient mice had higher bacterial burdens in blood and kidney (Figures 3E–H), indicating that IFI204 deficiency facilitates bacterial growth and dissemination.

### IFI204 Promotes Cytokines/Chemokines Production in Macrophages

**Figure 4:**
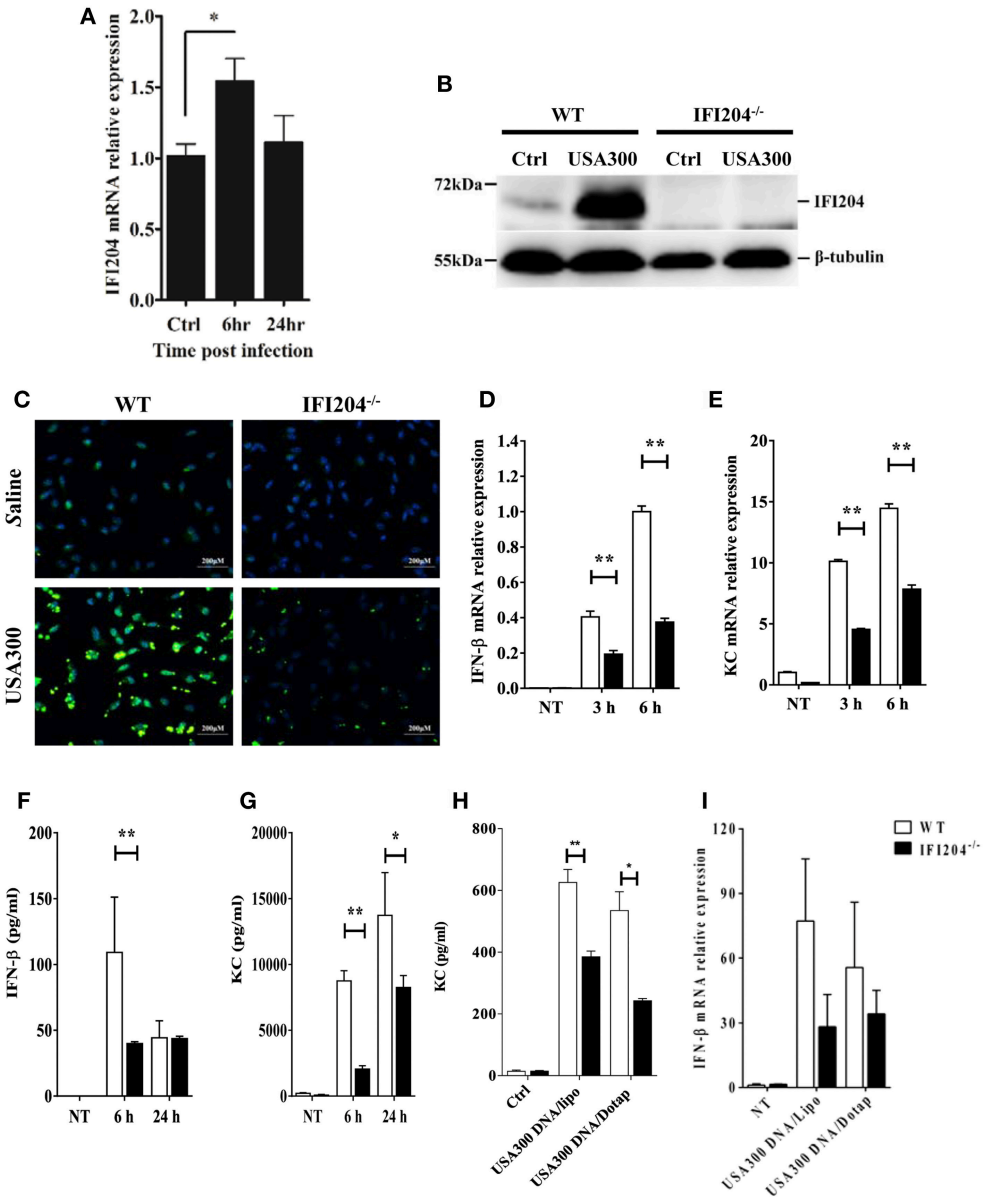
IFI204 is upregulated and promotes proinflammatory cytokines production in BMDM following *Staphyloccoccus* challenge. WT and IFI204^−/−^ BMDM were untreated or exposed to *Staphylococcus* at a MOI of 1:50 for indicated time. **(A)** IFI204 mRNA levels were measured by qRT-PCR. IFI204 protein in cells at 24 hpi was measured by Western blotting **(B)** and immunofluorescence microscopy **(C)**. Shown are representative images from two independent experiments. **(D–G)** Protein and/or mRNA levels of IFN-β and KC in BMDM were examined at the indicated time after infection. **(H,I)** WT and IFI204^−/−^ BMDM were transfected with *Staphylococcus*-derived genomic DNA using Lipofectamine 2000 or DOTAP. KC and IFN-β levels were quantified by ELISA or qRT-PCR. Data shown is representative of at least three independent experiments. All data are shown as mean ± SEM. Student's *t*-test was performed. ^*^p < 0.05; ^**^p < 0.01.

Next, we set out to characterize the inflammatory response in WT and IFI204-deficient macrophages *in vitro*. Bone marrow-derived macrophages (BMDM) derived from WT mice were infected with *Staphylococcus* at a multiplicity of infection (MOI) of 1:50. IFI204 mRNA and protein levels were significantly up-regulated by bacteria challenge (Figures 4A,B). We further examined IFI204 expression by immunofluorescence. IFI204 was detected in the nuclear of WT cells and absent from IFI204-deficient cells (Figure 4C). We next asked if IFI204 deficiency impair cytokines production in macrophages. *Staphylococcus* dramatically induced IFN-β and KC mRNA and protein expression in WT cells (Figures 4D–G). However, the levels of IFN-β and KC were significantly reduced in IFI204-deficient macrophages. IFI204 is characterized as a DNA sensor. A similar effect of IFI204 on KC and IFN-β production triggered by *Staphylococcus*-derived genomic DNA was found (Figures 4H,I). Collectively, these results provided *in vitro* evidence that IFI204 mediates cytokine secretion, which was consistent with the *in vivo* data.

### IFI204 Deficiency Suppresses STING-IRF3 and NF-κB Signaling

**Figure 5:**
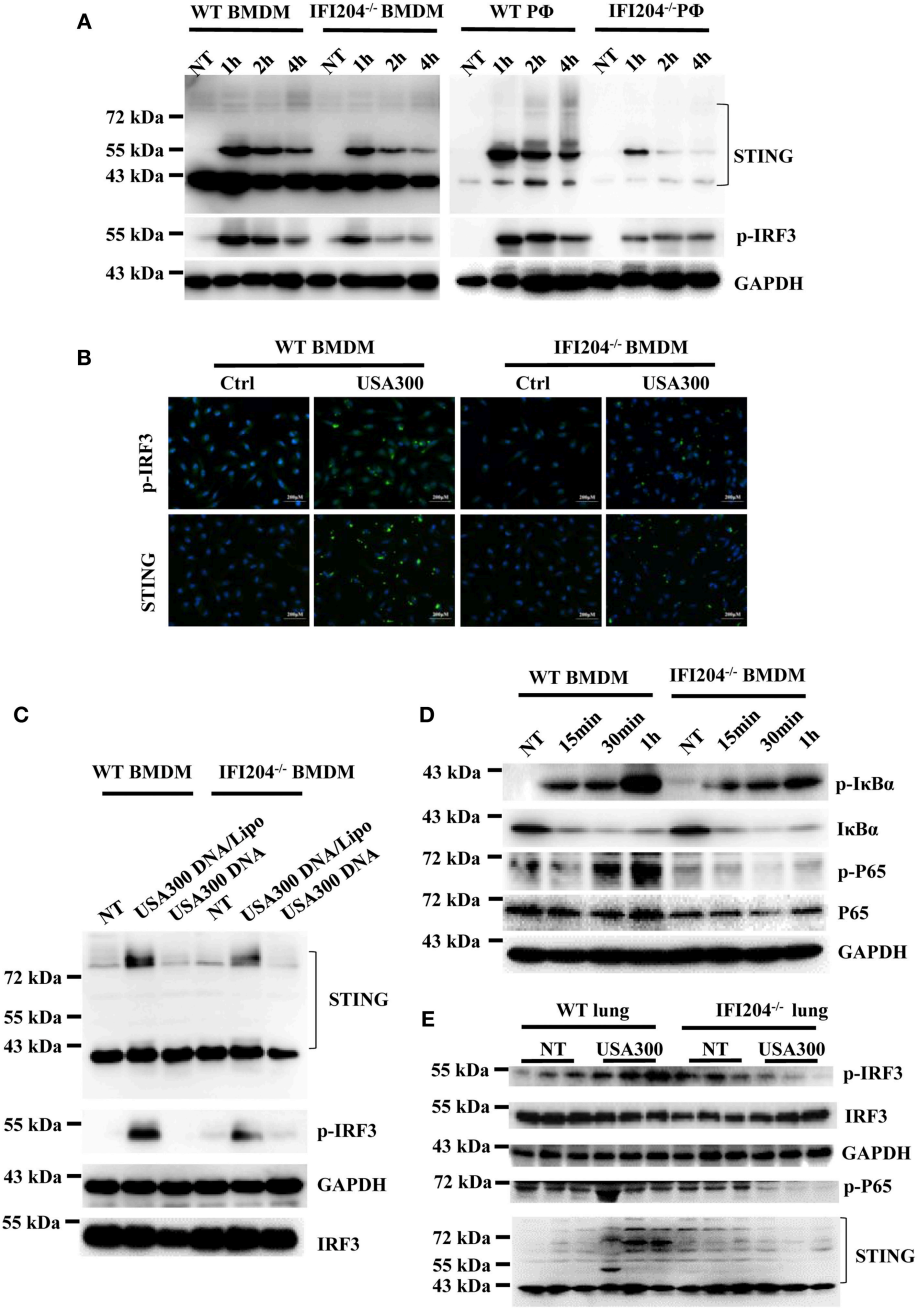
IFI204 deficiency suppresses *Staphylococcus*-induced activation of STING-IRF3 and NF-κB signaling. **(A)** WT and IFI204-deficient BMDM and peritoneal macrophages were untreated (NT) or exposed to *Staphylococcus* for the indicated times. The cell lysates were examined for expression levels of STING and phospho-IRF3 by Western blotting. The GAPDH served as a loading control. **(B)** WT and IFI204-deficient BMDM were exposed to *Staphylococcus* for 1 h. STING and phospho-IRF3 were analyzed by immunofluorescence. **(C)** WT and IFI204-deficient BMDM were incubated with USA300 DNA with or without Lipofectamine 2000 for 1 h. STING and phospho-IRF3 were examined by Western blotting. **(D)** WT and IFI204-deficient BMDM were exposed to *Staphylococcus* for the indicated times. Phospho-IκBα, IκBα, phospho-P65, P65, and GAPDH were examined by Western blotting. **(E)** WT and IFI204^−/−^ mice were infected intranasally with 1 × 10^8^ CFU of *Staphylococcus*. Lung tissues were collected and homogenized at 24 h postinfection, and then immunoblotting for Sting and phospho-IRF3, IRF3, GAPDH, P65, and STING.

To illustrate the signaling mechanism that results in cytokine decrease upon *Staphylococcus* infection in the absence of IFI204, IRF3 activation was assessed by Western blotting. The data showed that IRF3 was hypophosphorylated in IFI204-deficient BMDMs and peritoneal macophages compared with the control cells (Figure 5A). To further dissect the pathway, we analyzed STING, which plays a pivotal role in DNA-triggered induction of IFN-β (9). A significant decreased induction of STING was seen in IFI204-deficient macrophages (Figure 5A). The defect activation of STING and IRF3 were confirmed by immunofluorescence assay (Figure 5B). IFI204 deficiency also inhibited bacterial genomic DNA-triggered STING and IRF3 activation (Figure 5C). Moreover, NF-κB signaling was largely impaired in the absence of IFI204, showed by the reduced phosphorylation levels of p65 and IκBa in the treated IFI204-deficient macrophages (Figure 5D). Consistently, the lung tissues of IFI204-deficient mice challenged with bacteria showed a similar trend in significantly reduced STING-IRF3 and NF-κB activation compared with the controls (Figure 5E). Collectively, these results suggested that upon *Staphylococcus* challenge, IFI204 deficiency impaired the Sting-IRF3 and NF-κB pathways *in vitro* and *in vivo*.

### Neither KC Nor IFN-β Contributes to IFI204-Mediated Host Defense

**Figure 6:**
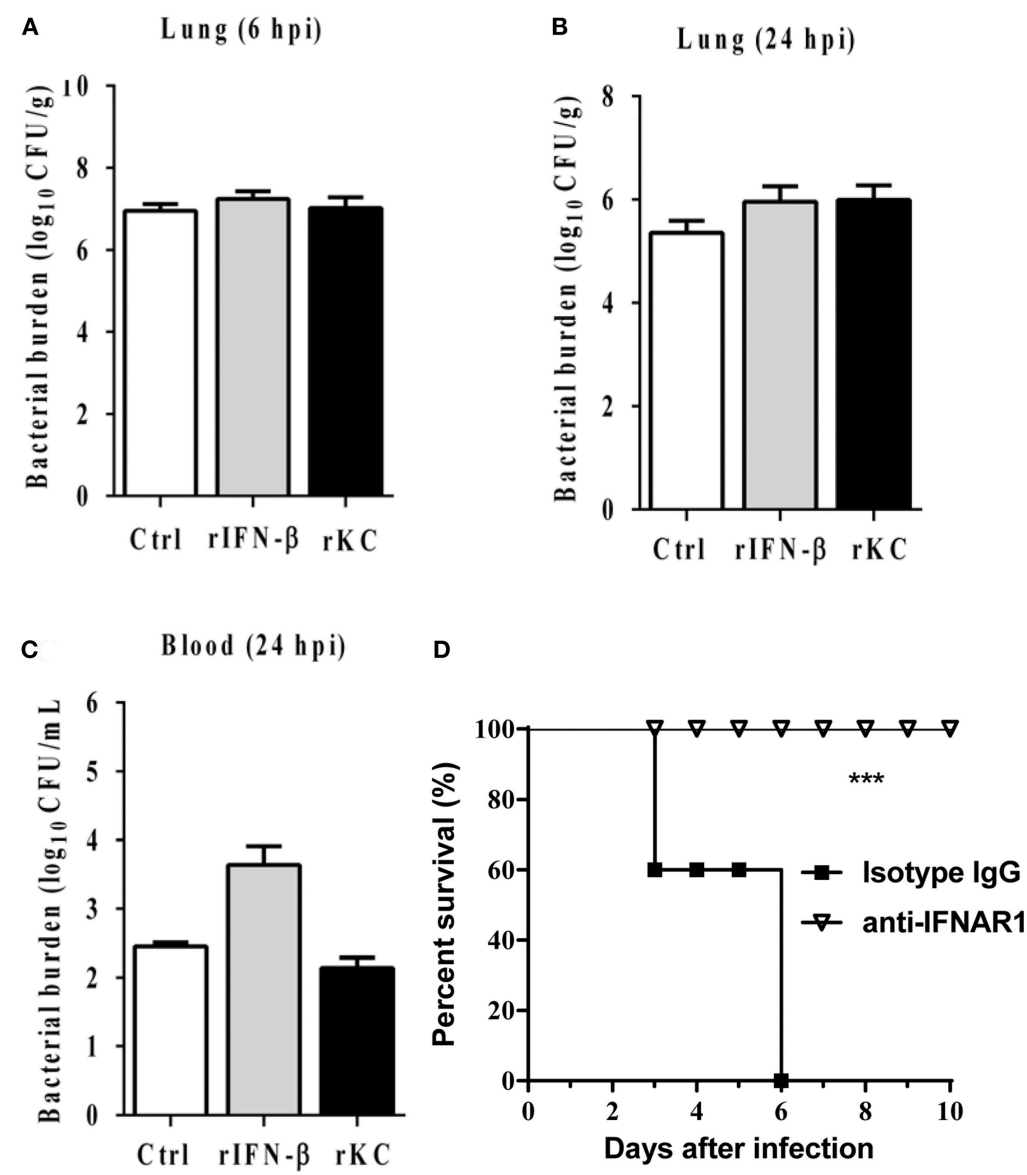
IFN-β and KC are dispensable for IFI204-mediated host defense. IFI204^−/−^ mice were i.p. injected recombinant KC or IFN-β (*n* = 10 each group) at a dose of 1.0 μg per mouse in 100 μL PBS on Day −1 and Day 0. The mice were infected intranasally with 1 × 10^8^ CFU of *Staphylococcus* on Day 0. **(A,B)** Homogenized lung tissues were subjected to plating serial dilution for bacterial loads at 6 hpi or 24 hpi. **(C)** Blood were subjected to plating serial dilution for bacterial loads at 24 hpi. All data are shown as mean ± SEM. Student's *t*-test was performed. **(D)** The mice were i.p. injected with 2.5 mg anti-mouse IFNAR1 neutralizing mAb or 2.5 mg IgG isotype control (*n* = 8 each group), 24 h later, mice were i.v. challenged with 2 × 10^8^ CFU of *Staphylococcus*. The animals were monitored daily for survival. ^***^p < 0.005.

Because cytokines generation and related proinflammatory signaling activation markedly attenuated in the absence of IFI204, we next examine whether exogenous administration of recombinant cytokines is able to rescue the susceptibility to infection in IFI204-deficient mice. IFI204-deficient mice were treated prophylactically (day −1 and day 0 of infection) with rKC or IFN-β and then infected with *Staphylococcus*. However, bacterial burdens in the lung tissue at 6 hpi were not inhibited by both recombinant proteins (Figure 6A). We further examined the effect of recombinant proteins at 24 hpi. A similar result was got (Figures 6B,C). Indeed, blockade of IFN signaling using anti-IFNAR1 MAb significantly decreased the mortality induced by *Staphylococcus* systemic infection (Figure 6D). Therefore, KC and IFN-β was not necessary for the protective effects of IFI204 during bacterial infection.

### IFI204 Deficiency Results in the Defect of Extracellular Trap-Mediated Bacteria Killing

**Figure 7:**
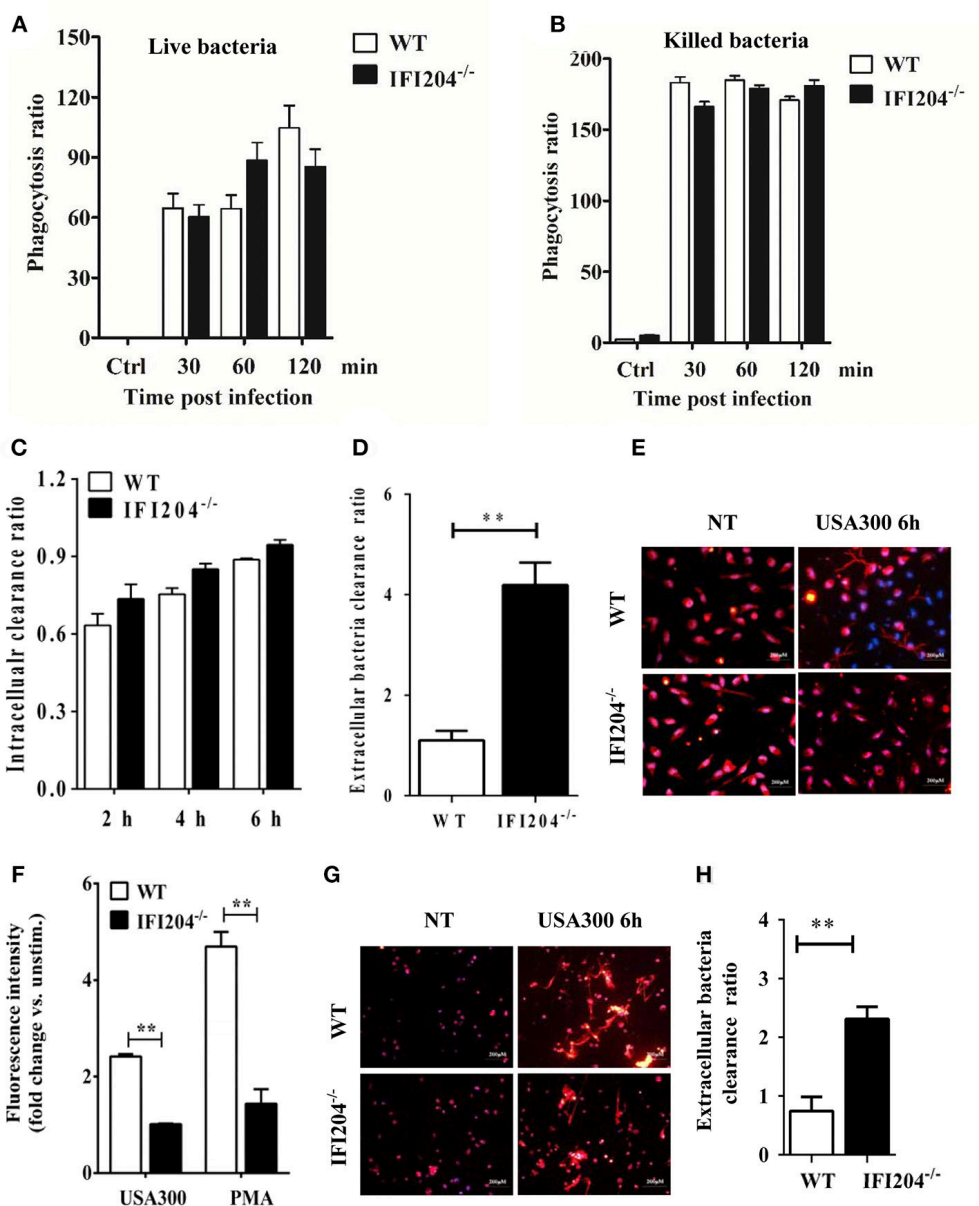
IFI204 promotes extracellular trap-mediated bacteria killing in macrophages and neutrophils. Macrophages were treated with FITC-labeled live **(A)** or killed bacteria **(B)**. The mean fluorescence intensity (MFI) of the FITC-positive cells were determined by flow cytometry. **(C)** Macrophages were infected with bacteria (MOI = 5) for 1 h. Non-engulfed bacteria were killed with gentamicin. The cells were lysed and intracellular bacterial were enumerated by plating on agar plates. **(D)** Extracellular bacterial killing capacity of macrophages were determined by assessing extracellular CFUs. **(E)** Macrophages were challenged with bacteria (MOI = 50) for 6 h. Representative microscopy pictures of NETs formation as indicated by SYTOX Orange. **(F)** Macrophages were stimulated with bacteria (MOI = 50) and PMA (100 nM). SYTOX Orange was added after 6 h and fluorescence was measured by spectrofluorometry. **(G)** Neutrophils were challenged with bacteria (MOI = 50) for 6 h. Representative microscopy pictures of NETs formation as indicated by SYTOX Orange. **(H)** Extracellular bacterial killing capacity of neutrophils were determined by assessing extracellular CFUs. All data are shown as mean ± SEM. Student's *t*-test was performed. ^**^p < 0.01.

Phagocytosis is a critical host defense mechanism used by macrophages. IFI204-deficient mice have elevated numbers of bacteria present in infected tissue, suggesting that these phagocytes are unable to effectively control bacterial multiplication in the absence of IFI204. We further evaluated the bacterial killing capacity of IFI204-deficient macrophages *in vitro*. Our results showed that IFI204-deficient macrophages internalized similar numbers of FITC-labeled live or heat-killed bacteria compared with WT macrophage (Figures 7A,B). Moreover, the gentamicin protection assay showed that the number of recovered viable bacteria was comparable in macrophages from both genotypes, indicating IFI204 has no effect on intracellular bacterial killing (Figure 7C). Generally speaking, *Staphylococcus* are extracellular bacteria that are eventually killed by phagocytes via multiple mechanisms besides phagocytosis. We further examined whether IFI204 deficiency impair extracellular killing capacity by enumerating bacteria in supernatants at 6 hpi. The results showed that IFI204 deficiency inhibited the extracellular killing capacity of macrophage (Figure 7D). Extracellular trap (ET) provides an extracellular site for microbial killing in the innate immune defense. To investigate the effect of IFI204 deficiency on ET formation, we stained macrophages with SYTOX Orange, a non-permeable dye that stains nucleic acid, a primary component of ET. Interestingly, MET formation was markedly decreased in IFI204-deficient macrophage vs. WT macrophages (Figure 7E). To quantify MET formation, we analyzed extracellular DNA content in the supernatants. A reduction of extracellular DNA was seen in IFI2014-deficient macrophages compared with WT cells (Figure 7F). Moreover, IFI204 deficiency impaired PMA-induced extracellular DNA release. Because an initial description of ET appeared in neutrophil, another type of phagocyte that play important roles in host's defense against infection, we further examined if there is a defect of ET formation in IFI204-deficient neutrophils. A similar defect of NET formation was observed in bacteria-infected IFI204-deficient neutrophils vs. WT neutrophils (Figure 7G). Correspondingly, IFI204-deficient neutrophils were incapable of killing extracellular bacteria compared with WT neutrophils (Figure 7H). Hence, these data suggested that IFI204 deficiency leads to a defect in extracellular bacterial killing by impairing ET formation in phagocytes.

### Bone Marrow Transplantation Restore Bacteria Killing in IFI204-Deficient Mice

**Figure 8:**
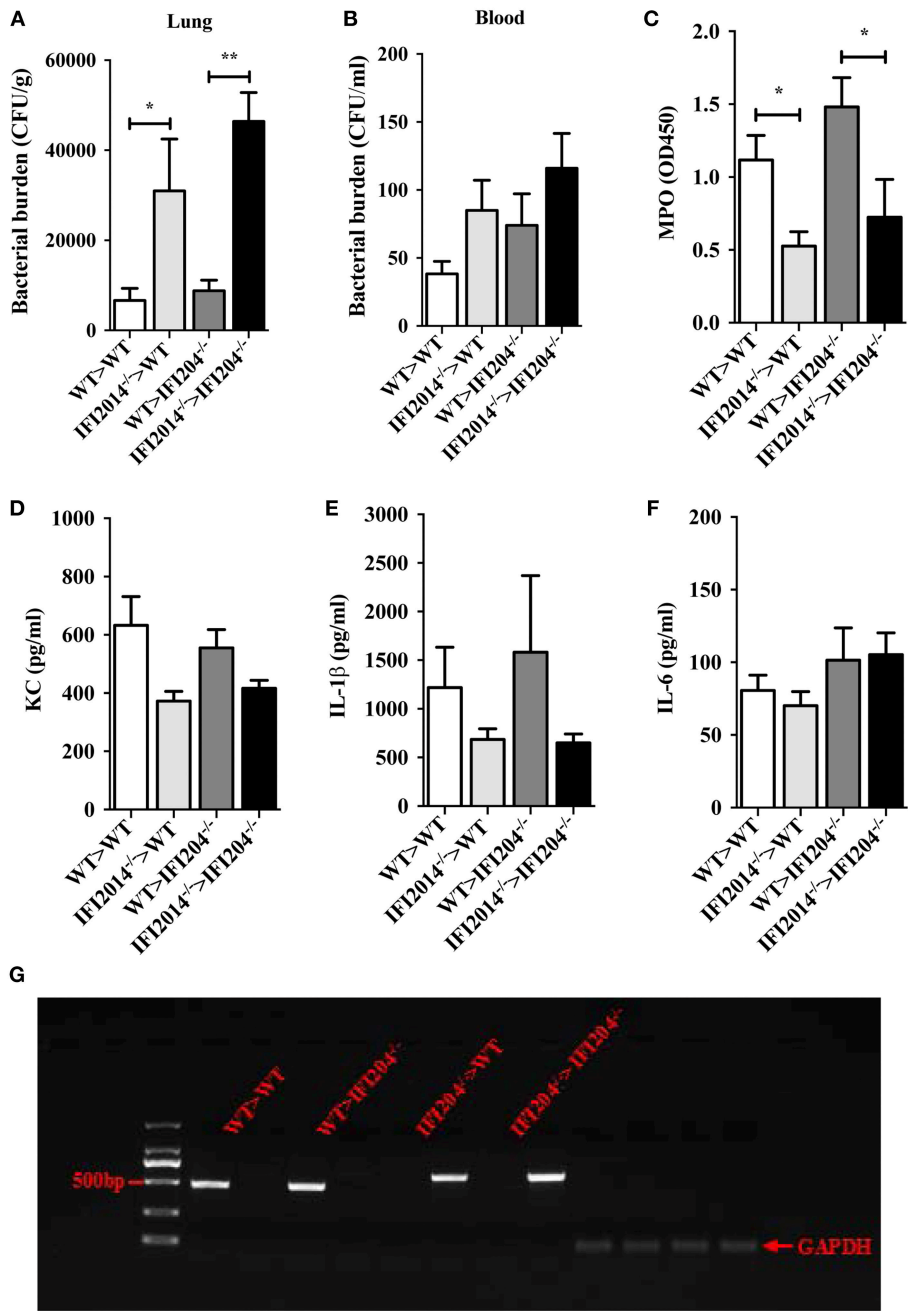
Adoptive transfer of WT BM cells protects IFI204-deficient mice against *Staphylococcus* infection. Bone marrow cells isolated from WT or IFI204^−/−^ mice were adoptively transferred to irradiated IFI204^−/−^ or WT recipient mice. The recipient mice were then infected nasally with *Staphylococcus* (1 × 10^8^ CFU, *n* = 4–6 each group) for 24 h. **(A,B)** Homogenized lung tissue and blood were subjected to plating serial dilution for bacterial loads. **(C)** Neutrophil activity in lung was determined by the amount of MPO. **(D–F)** The release of KC, IL-6, IL-1β in lung were determined by ELISA. **(G)** Efficient reconstitution by donor bone marrow cells was confirmed by PCR for the IFI204 gene in splenocytes. All data are shown as mean ± SEM. Student's *t*-test was performed. ^*^p < 0.05; ^**^p < 0.01.

To further substantiate the pivotal role of IFI204 in enhancing extracellular bacteria killing, we further examined if transplantation of WT bone marrow (BM) rescue bacterial killing defect in IFI204-deficient mice. WT or IFI204-deficient recipient mice were lethally irradiated and injected with BM cells from WT or IFI204-deficient donors. Eight weeks after transplantation, those mice were inoculated with *Staphylococcus*, and bacterial burdens in the lung and blood were determined. Similar to WT recipient mice that received WT BM, IFI204-deficient recipient mice that received WT BM had less bacterial burden (Figures 8A,B). Conversely, WT recipient mice that received IFI204-deficient BM had more bacterial burden in lung tissue, identical to that observed in IFI204-deficien recipient mice that received IFI204-deficient BM. The similar pattern was observed in the activity of MPO (Figure 8C), one granule enzyme which plays an important role in neutrophil antimicrobial responses and is required for neutrophil extracellular trap formation (10). The proinflammatory cytokines (KC and IL-1β) in lung showed slight similar patterns (Figures 8D–F). Depletion of circulating cells and reconstitution with donor cells was comfirmed (Figure 8G). Together, the results indicate that the protective effect of IFI204 against *Staphylococcus* infection is dependent on the ability of IFI204 enhancing phagocyte killing capacity.

## Discussion

*Staphylococcus aureus* infections are usually persistent and hard to eradicate. Development of new therapeutic strategies to combat *Staphylococcus* infections requires deeper understanding of molecular mechanisms underlying phagocyte functions in antibacterial defense. Recently, several studies including ours showed that murine IFI204 or human ortholog IFI16 detects cytosolic bacterial DNA for the type I IFN response or cell death *in vitro* (5–8). Hence, IFI204/IFI16 is implicated in sensing intracellular bacterial infection. Generally speaking, *Staphylococcus* is extracellular bacteria. Using IFI204-deficient mice, we demonstrate that IFI204 promotes host survival and bacterial clearance during *Staphylococcus* pulmonary and systemic infection. Therefore, we defining a novel role for IFI204 in host defense against extracellular bacterial infection.

To identify the potential mechanisms underlined IFI204-mediated defense, we set out to characterize the inflammatory response. IFI204 deficiency leads to an inferior production of cytokines/chemokines in the lung following *Staphylococcus* infection. Due to IFI204 mainly locating in the recruited inflammatory cells, bone marrow-derived macrophages were used for determine IFI204-mediated inflammatory response. Consistent with *in vivo* data, IFI204 deficiency not only impairs cytokines production in macrophages, but also inhibits bacteria-induced STING-IRF3 and NF-κB activation. IFI204/IFI16 is extensively characterized as a DNA sensor, which detects cytosolic DNA derived from virus, bacteria, even host DNA (11). In the presence of intracellular DNA, IFI204/IFI16 interacts with STING to induce TBK1-dependent IFN-β responses. Using *Staphylococcus*-derived genome DNA, we also showed that cytosolic DNA activates the STING-IRF3 pathway, and promotes IFN-β and KC productions. Hence, it is quite possible that IFI204 serve as DNA sensor to trigger inflammatory responses during *Staphylococcus* infection.

IFN-β is produced during viral infections and is responsible for defense against viruses. IFN-β also induced by *Staphylococcus*. However, the role of IFN-β during *Staphylococcus* infection varies. It can be both beneficial (12, 13) and detrimental (14–16) to the host, probably depending on the experimental design. We hypothesized that the defect of IFN-β production may leads to susceptibility to infection in the absence of IFI204. Unexpectedly, administration of recombinant IFN-β even promotes bacteria proliferation in IFI204-deficient mice. Moreover, blockade of IFN signaling significantly decreased the mortality induced by *Staphylococcus* systemic infection. Hence, it suggests that IFN-β was not only unnecessary for the protective effects of IFI204, but also detrimental to the host during *Staphylococcus* infection. Our data also showed that KC production is attenuated in IFI204-deficient mice and macrophages. KC has been shown to have a critical role in protective responses to *Staphylococcus* infection. However, administration of recombinant mouse KC is still unable to restrict *Staphylococcus* multiplication in IFI204-deficient mice.

Phagocytosis, a process by which myeloid cells such as macrophages and neutrophils internalize and kill microorganisms, is the critical host innate defense mechanism. Our results showed that IFI204-deficient macrophages internalized similar numbers of FITC-labeled live or heat-killed bacteria compared with WT macrophages. Moreover, WT and IFI204-deficient macrophages exhibit a comparable capacity for intracellular killing. Because bacterial phagocytosis in IFI204-deficient macrophages was not impaired, it is possible that IFI204 participates in the regulation of phagocytosis-independent bacterial killing such as the process mediated by extracellular trap. Extracellular trap was first described in neutrophils as the released of web-like structures after stimulation with Gram-positive or Gram-negative bacteria. Increased evidences showed that ET is not formed exclusively by neutrophils but also by other innate myeloid cells including macrophage (17), basophils (18), eosinophil (19), and mast cells (20) response to microbes. Activated innate myeloid cells release these structures composed of decondensed chromatin and antimicrobial proteins that trap and inhibit a broad range of microbes. Compared with control cells, both IFI204-deficient macrophages and neutrophils are incapable of killing extracellular bacteria, as well as reduce the release of extracellular DNA. Moreover, transplantation of WT bone marrow rescued bacterial killing defect in IFI204-deficient mice. Hence, our results indicate that the protective effect of IFI204 against *Staphylococcus* infection is dependent on the ability of IFI204 enhancing phagocyte killing capacity by promoting ET formation.

Some other pattern recognition receptor including TLR4 (21–23), TLR7/8 (24, 25), TLR2 (23, 26, 27), TLR6 (28), TLR9 (29), lectin receptors Mincle and CLEC5A (30–32), Fc receptors FcαRI and FcγRIIIb (33, 34) were implicated in neutrophil extracellular trap formation. TLR2/4 was found to modify NET formation in response to *Staphylococcus* infection but not to PMA stimulation (23). Interestingly, we observed that IFI204 impact both pathogen and PMA-induced extracellular DNA release. Several virulence factors of *Staphylococcus* were reported to elicit ET formation, including leukotoxin (35), leukocidins (36), phenol-soluble modulin α (PSMα) (37), and protein A (38). Given the fact that IFI204 mediates PMA-induced extracellular DNA release, we speculate that IFI204 probably promotes extracellular bactericidal activity independent of DNA recognition. While understanding the underlying mechanism of IFI204 regulating ET awaits further investigation, this study extends our understanding the biological function of IFI204 in host innate immune response.

In summary, our studies demonstrate IFI204 is essential for host defense against *Staphylococcus* infection *in vivo*. IFI204 promotes bacteria eradication and inflammation response. However, inflammation response does not contribute to IFI204-mediated protection. Moreover, we provide evidence that IFI204 plays a role in extracellular bactericidal activity of phagocytes through enhancing extracellular trap formation. These observations document a novel and physiologically important role for IFI204 in host defense against extracellular bacterial infection.

## Data Availability

All datasets generated for this study are included in the manuscript and/or the supplementary files.

## Ethics Statement

All animal studies were conducted according to experimental practices and standards approved by the Animal Welfare and Research Ethics Committee at Jilin University (No. 20150601).

## Author Contributions

WC and Y-JY: designed experiments. WC, S-XY, F-HZ, and X-JZ: performed the experiments. WC and S-XY: analyzed the data. Y-JY: wrote the manuscript. W-YG, K-YL, Z-ZL, and W-YH: read the manuscript.

### Conflict of Interest Statement

The authors declare that the research was conducted in the absence of any commercial or financial relationships that could be construed as a potential conflict of interest.

## Abbreviations

IFI204: interferon-inducible protein 204
IFI16: interferon gamma inducible protein 16
*Staphylococcus*: *Staphylococcus aureus*
MOI: multiplicity of infection
BMDM: bone marrow-derived macrophage
PMA: phorbol 12-myristate 13-acetate
PRR: pattern recognition receptor
TLR: toll-like receptor
STING: stimulator of interferon genes
CFU: colony-forming unit
H&E: hematoxylin and eosin
rIFN-β: recombinant IFN-β
rKC: recombinant KC.
